# Altered Spatial and Temporal Gait Parameters in Mice Infected with Ross River Virus

**DOI:** 10.1128/mSphere.00659-21

**Published:** 2021-09-29

**Authors:** Eranga Abeyratne, Ronak Reshamwala, Todd Shelper, Xiang Liu, Ali Zaid, Suresh Mahalingam, Adam Taylor

**Affiliations:** a Menzies Health Institute Queensland, Griffith University, Gold Coast Campus, Southport, QLD, Australia; b Global Virus Network Centre of Excellence in Arboviruses, Griffith University, Gold Coast Campus, Southport, QLD, Australia; c School of Pharmacy and Medical Sciences, Griffith University, Gold Coast Campus, Southport, QLD, Australia; d Griffith Institute for Drug Discovery, Griffith University, Gold Coast Campus, Southport, QLD, Australia; e Clem Jones Centre for Neurobiology and Stem Cell Research, Griffith University, Gold Coast Campus, Southport, QLD, Australia; University of Maryland School of Medicine

**Keywords:** alphavirus, Ross River virus, Barmah Forest virus, gait, infectious arthritis, arbovirus, mouse model, running, stride

## Abstract

Infection with mosquito-borne arthritogenic alphaviruses, such as Ross River virus (RRV) and Barmah Forest virus (BFV), can lead to long-lasting rheumatic disease. Existing mouse models that recapitulate the disease signs and immunopathogenesis of acute RRV and BFV infection have consistently shown relevance to human disease. However, these mouse models, which chiefly model hindlimb dysfunction, may be prone to subjective interpretation when scoring disease. Assessment is therefore time-consuming and requires experienced users. The DigiGait system provides video-based measurements of movement, behavior, and gait dynamics in mice and small animals. Previous studies have shown DigiGait to be a reliable system to objectively quantify changes in gait in other models of pain and inflammation. Here, for the first time, we determine measurable differences in the gait of mice with infectious arthritis using the DigiGait system. Statistically significant differences in paw area and paw angle were detected during peak disease in RRV-infected mice. Significant differences in temporal gait parameters were also identified during the period of peak disease in RRV-infected mice. These trends were less obvious or absent in BFV-infected mice, which typically present with milder disease signs than RRV-infected mice. The DigiGait system therefore provides an objective model of variations in gait dynamics in mice acutely infected with RRV. DigiGait is likely to have further utility for murine models that develop severe forms of infectious arthritis resulting in hindlimb dysfunction like RRV.

**IMPORTANCE** Mouse models that accurately replicate the immunopathogenesis and clinical disease of alphavirus infection are vital to the preclinical development of therapeutic strategies that target alphavirus infection and disease. Current models rely on subjective scoring made through experienced observation of infected mice. Here, we demonstrate how the DigiGait system, and interventions on mice to use this system, can make an efficient objective assessment of acute disease progression and changes in gait in alphavirus-infected mice. Our study highlights the importance of measuring gait parameters in the assessment of models of infectious arthritis.

## INTRODUCTION

Infectious arthritis manifests as joint pain, arthralgia, and inflammation of the joints caused by pathogens such as fungi, bacteria, and viruses. The clinical manifestations of infectious arthritis are similar to those of rheumatoid arthritis. Indeed, there is significant overlap in the proinflammatory gene expression profiles of certain forms of infectious arthritis and rheumatoid arthritis (1). Pathogens such as human immunodeficiency virus, adenovirus, and the bacteria *Pasteurella* and *Rickettsia* can all cause rheumatic manifestations in infected patients (2–4). However, the most common causes of infectious arthritis worldwide are arthropod-borne arthritides, including arthritogenic alphaviruses and the bacterium *Borrelia*, transmitted by infected mosquitoes and ticks, respectively.

Infection with mosquito-borne arthritogenic alphaviruses, such as chikungunya virus, Ross River virus (RRV), and Barmah Forest virus (BFV), can lead to rheumatic disease. Symptoms of infection are similar among arthritogenic alphaviruses and include fever, rash, lethargy, polyarthritis, and muscle pain. Arthritogenic alphaviruses are globally widespread and capable of causing explosive outbreaks of chronic, highly debilitating musculoskeletal disease. Infected patients can experience relapses of joint pain or chronic arthritis or myalgia that can last for years. The extremely debilitating, chronic nature of arthritogenic alphavirus disease contributes most significantly to the overall disease burden. No commercial vaccines, antivirals, or targeted therapeutics are currently available to treat alphavirus disease. RRV and BFV are arthritogenic alphaviruses endemic to Australia, where they are responsible for on average 6,000 notifications of disease per year (http://www9.health.gov.au/cda/source/cda-index.cfm). The largest recorded outbreak of RRV in Australia saw 9,539 cases in 2015, almost double the national average (http://www9.health.gov.au/cda/source/cda-index.cfm). The 2017 RRV outbreak in Victoria, Australia, was a concerning development, with more than 1,000 cases recorded in just 6 weeks and potentially the first detection of RRV in metropolitan Melbourne. RRV is also endemic to Papua New Guinea, and recent studies provide serological evidence of RRV circulating in the neighboring Pacific Islands (5, 6).

Mouse models that recapitulate the disease signs and immunopathogenesis of RRV and BFV infection have been developed to dissect virus-induced inflammation (7). These models have consistently shown relevance to human disease, with significant overlap in both the soluble and cellular immune factors identified throughout the course of disease in infected humans and mice (8). They have proven to be a useful platform to develop and test novel therapeutics (9, 10). The C57BL/6 mouse models of acute RRV and BFV diseases are both models of hindlimb weakness followed by recovery (8, 11). For the RRV model, 21-day-old C57BL/6 mice are subcutaneously inoculated and develop viremia that is detected at ∼5 to 6 days postinfection (dpi). From the blood, RRV is able to replicate at secondary sites of infection, including skeletal muscles and joints. Disease onset is observed by 4 to 5 dpi with signs such as ruffled fur, hunched posture, and weight loss (12). Disease signs peak at approximately 10 dpi with severe hindlimb dysfunction, loss of grip strength, and severe weight loss. The RRV-induced inflammatory response results in severe myositis and bone loss in mice at peak disease (13, 14). At 15 to 21 dpi, the disease begins to resolve, with mice regaining hindlimb function and gaining weight. By 30 dpi, mice have fully recovered from infection and show no signs of disease. In comparison to the RRV model, BFV-infected mice display moderate disease signs at peak disease, with only slight hindlimb weakness (8). Although BFV still has tropism for muscle and joint tissues, BFV does not replicate to as high a titer as RRV and induces only a moderate inflammatory response (8). Both models are, however, prone to subjective interpretation when scoring disease and often rely on labor-intensive blinding to limit operator bias.

The increasing incidence and severity of infectious arthritic disease highlight the urgent need to develop targeted treatments and control measures toward emerging infectious arthritides (15, 16). Effective models of infectious arthritis are therefore vital for understanding the pathophysiology of infection and thus assessing the therapeutic potential of novel treatments. Multiple murine models of infectious arthritis, including the C57BL/6 models of RRV and BFV infection, monitor gait to some extent when determining clinical scores (17). However, these models, on the whole, rely heavily on subjective semiquantitative measures of gait, for example, the severity of lethargy, hindlimb weakness, or lameness. This type of analysis is open to individual interpretation and requires experienced users.

The DigiGait system provides an objective assessment of movement, behavior, and gait dynamics in mice and small animals. Previous studies have shown DigiGait to be a reliable system to objectively quantify behavioral changes and changes in gait in models of pain and inflammation, including carrageenan-induced inflammation (18, 19). Here, for the first time, we determine objective differences in the gait of mice with infectious arthritis using the DigiGait system.

## RESULTS

### Arthritogenic alphavirus-infected mice require intervention to run on the DigiGait apparatus during peak acute disease.

**FIG 1:**
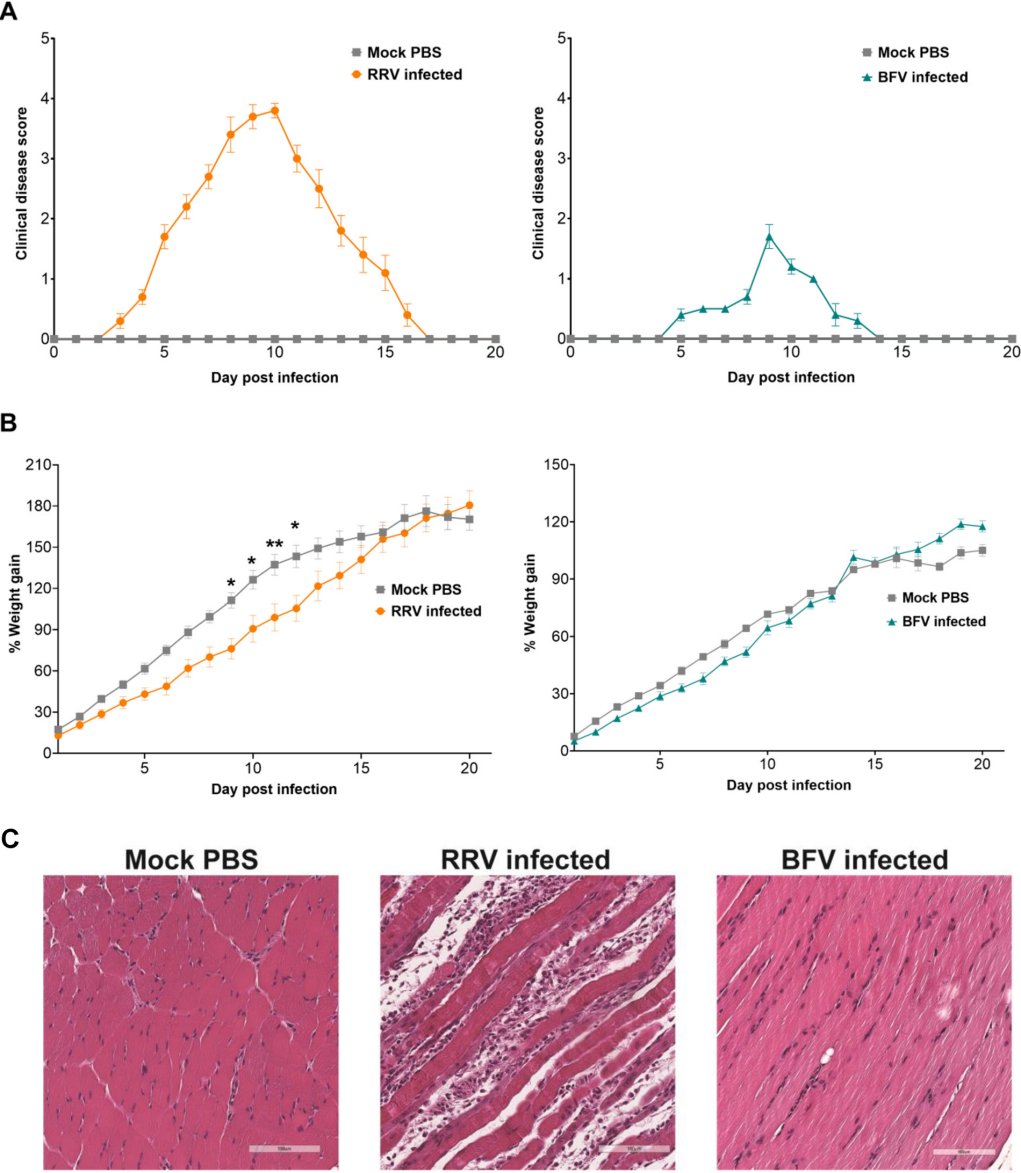
Clinical disease signs, weight gain, and muscle tissue pathology in RRV- and BFV-infected mice. Twenty-one-day-old C57BL/6 female mice (*n *= 5) were inoculated subcutaneously with 10^4^ PFU of RRV or BFV, and mock-infected mice were inoculated with PBS diluent alone. Mice were weighed and scored for disease daily. Mice were scored according to hindlimb strength and the onset of hindlimb dysfunction, as described in Materials and Methods. (A and B) The clinical disease score (A) and percent weight gain (B) of RRV- and BFV-infected mice were determined. Mock-infected mice showed no disease signs for the duration of the experiment. (C) At 10 dpi, quadriceps were collected, fixed in 4% paraformaldehyde, paraffin embedded, cut into 5-μm sections, and stained with hematoxylin and eosin. Images are at a ×200 magnification and are representative of results for 5 mice. Values are the means ± standard errors of the means. Mouse weight was analyzed using two-way analysis of variance (ANOVA) with a Bonferroni *post hoc* test (*, *P < *0.05; **, *P < *0.01).

The established murine models of acute RRV and BFV disease rely on the observable development of musculoskeletal pathology. Disease severity in both models is principally measured by subjective assessment of hindlimb weakness. RRV- and BFV-infected mice also exhibit reduced weight gain compared to mock-infected mice. To assess the differences in the gait of mice infected with arthritogenic alphaviruses, C57BL/6 mice were acclimatized to running on the DigiGait apparatus and infected subcutaneously with either 10^4^ PFU of RRV or 10^4^ PFU of BFV as described previously (8, 20). Videos of mice were recorded daily using the DigiGait apparatus with mice running at 25 cm/s for a minimum of 10 consecutive strides. Mice ran at 25 cm/s for a maximum of 1 min. Mice were scored for the development of disease manifestations using the established clinical scoring matrix. As reported previously, RRV-infected mice developed observable clinical signs of disease by 3 days postinfection (dpi) (Fig. 1A) (20). Disease signs peaked at 10 dpi, with RRV-infected mice displaying severe hindlimb dysfunction and lethargy. RRV-infected mice showed significantly reduced weight gain between 9 dpi and 12 dpi (Fig. 1B). By 16 dpi, RRV-infected mice had gained ∼160% of their mean starting body weight, similar to mock-infected mice. Hindlimb function progressively improved following peak disease, with no observable disease signs by 17 dpi in RRV-infected mice. BFV-infected mice presented with disease signs at 5 dpi (Fig. 1A). BFV disease peaked at 9 dpi, and by 14 dpi, clinical signs of disease were undetectable by observation. BFV-infected mice showed a moderate reduction in weight gain compared with mock-infected mice, as seen previously (Fig. 1B) (8). Histopathological analysis confirmed that the quadriceps of RRV-infected mice had greater signs of inflammation and muscle tissue damage than those of BFV-infected mice at 10 dpi (Fig. 1C).

**FIG 2:**
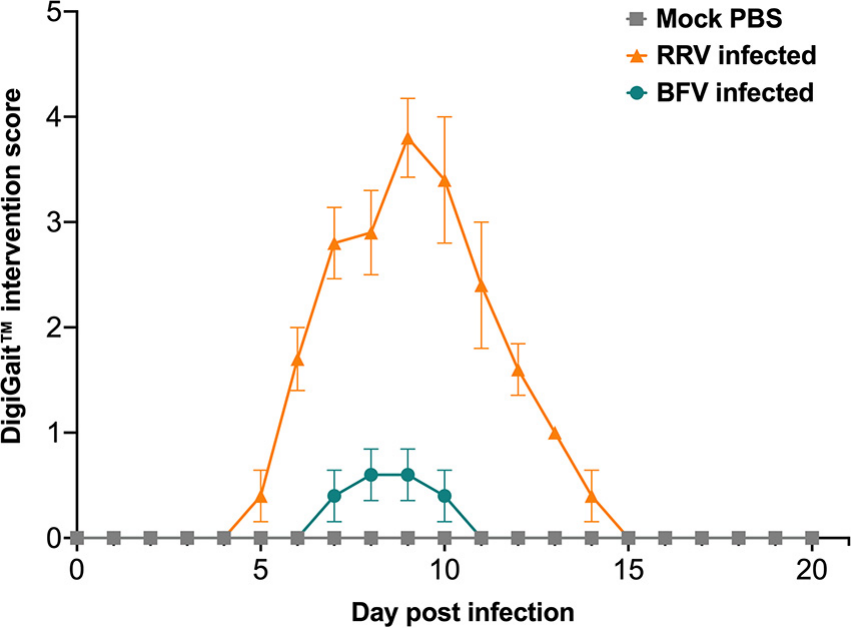
Degree of intervention required for infected mice to run at 25 cm/s. RRV-, BFV-, and mock-infected mice (*n *= 5) were scored according to the degree of intervention required to stimulate running at 25 cm/s on the DigiGait treadmill, as described in Materials and Methods. Mock-infected mice required no intervention to reach a running speed of 25 cm/s for the duration of the experiment. Values are the means ± standard errors of the means.

To ensure DigiGait video quality while minimizing the impact of extended periods of movement on the mice, mouse movement was recorded in the same field of view for at least 10 consecutive, repeatable gait cycles at 25 cm/s. During the development of disease, RRV- and BFV-infected mice demonstrated various degrees of ability in reaching a speed of 25 cm/s for 10 consecutive strides. Mice were therefore scored daily for their ability to reach a speed of 25 cm/s with and without intervention, as described in Materials and Methods. At between 6 and 13 dpi, all RRV-infected mice required some level of intervention to reach a speed of 25 cm/s (Fig. 2). At 9 dpi, all RRV-infected mice required at least 2 to 5 min of habituation to the apparatus followed by >5 on/off cycles of the DigiGait treadmill to ultimately reach 25 cm/s. One RRV-infected mouse found it extremely difficult to reach a speed of 25 cm/s, and movement at a lower speed (15 to 20 cm/s) was required before reaching 25 cm/s at 9 and 10 dpi. At between 7 and 10 dpi, a small number of BFV-infected mice required minimal intervention (<5 on/off cycles of the DigiGait treadmill) to reach 25 cm/s (Fig. 2).

### DigiGait analysis of Ross River virus-infected mice reveals differences in spatial and temporal gait parameters during peak acute disease.

**FIG 3:**
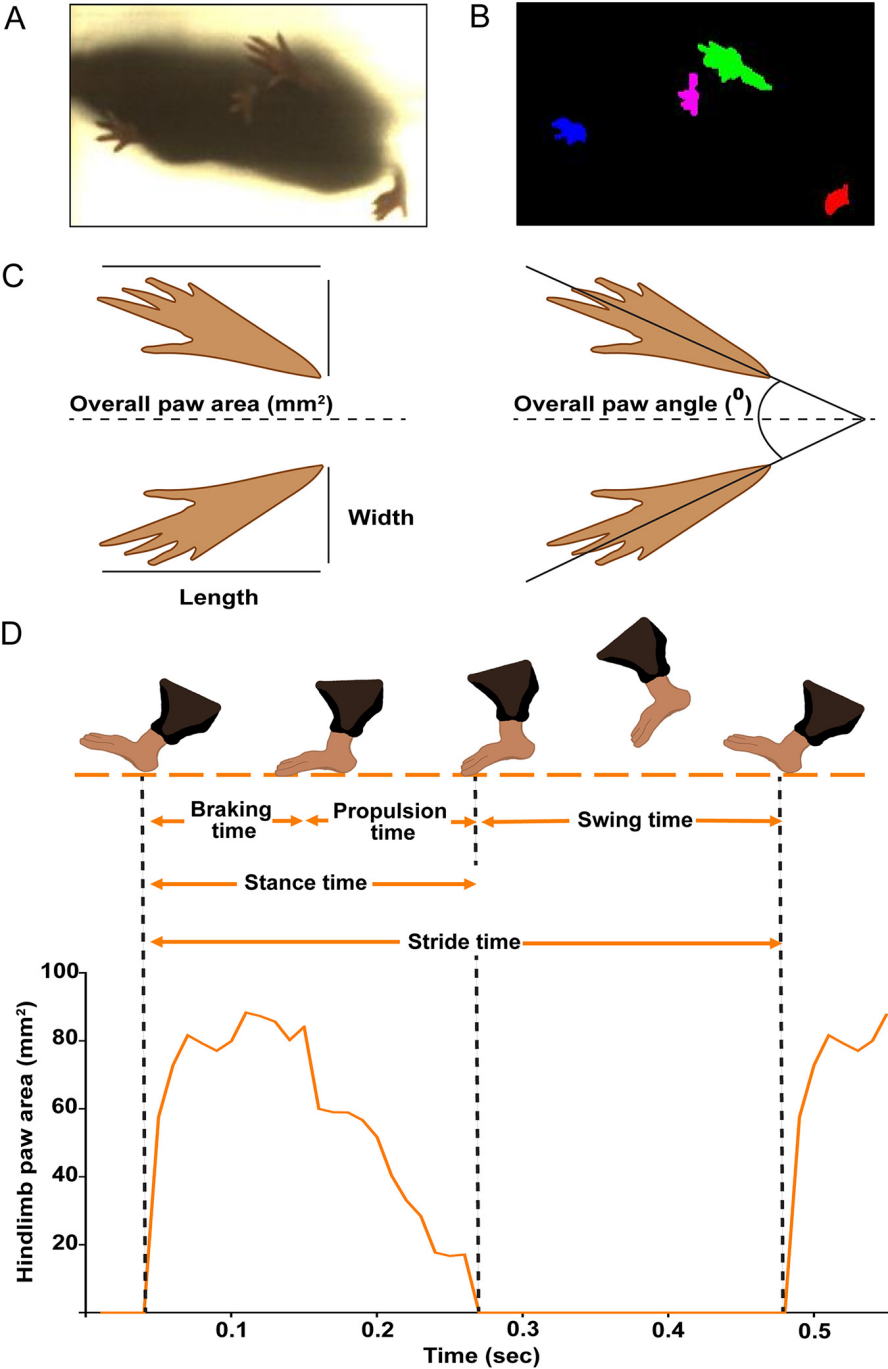
DigiGait imaging system and gait parameters. (A) Ventral image of a mouse in the DigiGait running chamber taken by a high-speed video camera mounted below a transparent treadmill belt. (B) Digital paw prints generated by the DigiGait ventral plane imaging technology. (C) Diagrammatic representations of the digital paw prints used to measure the spatial gait parameters overall hindlimb paw area and overall hindlimb paw angle. (D) Diagrammatic representations of temporal gait parameters defined by paw area contact with the belt. Stride time, the time for one limb to complete a stride cycle; stance time, the time in which the paw remains in contact with the belt; swing time, the time during which the paw is not in contact with the belt and moving forward; brake time, the time between initial paw contact and maximum paw contact with the belt; propulsion time, the time between maximum paw contact and the start of the swing phase.

**FIG 4:**
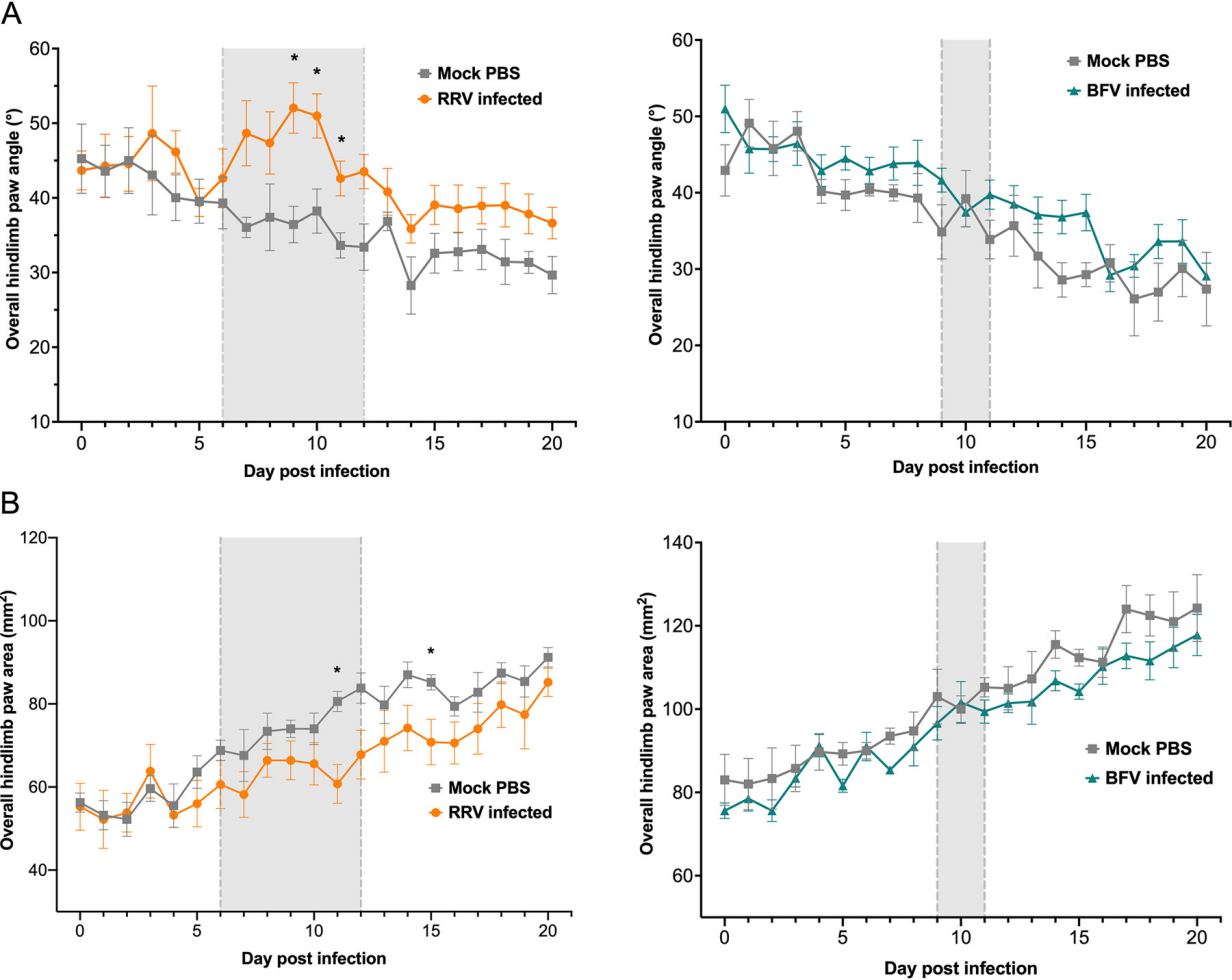
RRV-infected mice show significant changes in spatial gait parameters at peak acute disease compared to mock-infected mice. Hindlimb overall paw angle (A) and area (B) were analyzed in RRV-, BFV-, and mock-infected mice (*n *= 5) using the DigiGait apparatus. Values are the means ± standard errors of the means. Hindlimb overall paw area and angle were analyzed using the nonparametric Mann-Whitney test (*, *P < *0.05). The gray background bound by gray dashed lines indicates the period during which RRV-infected mice received an average clinical disease score of ≥2 and BFV-infected mice received an average clinical disease score of ≥1.

Previous studies demonstrate the utility of the DigiGait system to objectively quantify changes in gait in diverse models of pain and inflammation (18, 19). Changes in the gaits of RRV- and BFV-infected mice were quantified using the DigiGait imaging system (Mouse Specifics Inc.), as described in Materials and Methods (Fig. 3). In mock-infected mice, the mean overall hindlimb paw angle decreased from ∼45° at 0 dpi to ∼30° at 20 dpi (Fig. 4A). This change likely reflects the age and weight gain of these juvenile mice over the course of the experiment. In RRV-infected mice, the mean overall hindlimb paw angle increased to 52.04° at 9 dpi (Fig. 4A). A significant increase in the overall hindlimb paw angle was measured in RRV-infected mice at 9, 10, and 11 dpi, corresponding to peak disease, compared to mock-infected mice. No significant difference in overall hindlimb paw angle was detected between mock- and BFV-infected mice (Fig. 4A). RRV-infected mice also showed a significantly reduced overall hindlimb paw area at 11 and 15 dpi compared to mock-infected mice (Fig. 4B). No difference in overall hindlimb paw area was detected between mock- and BFV-infected mice over the course of the infection (Fig. 4B).

**FIG 5:**
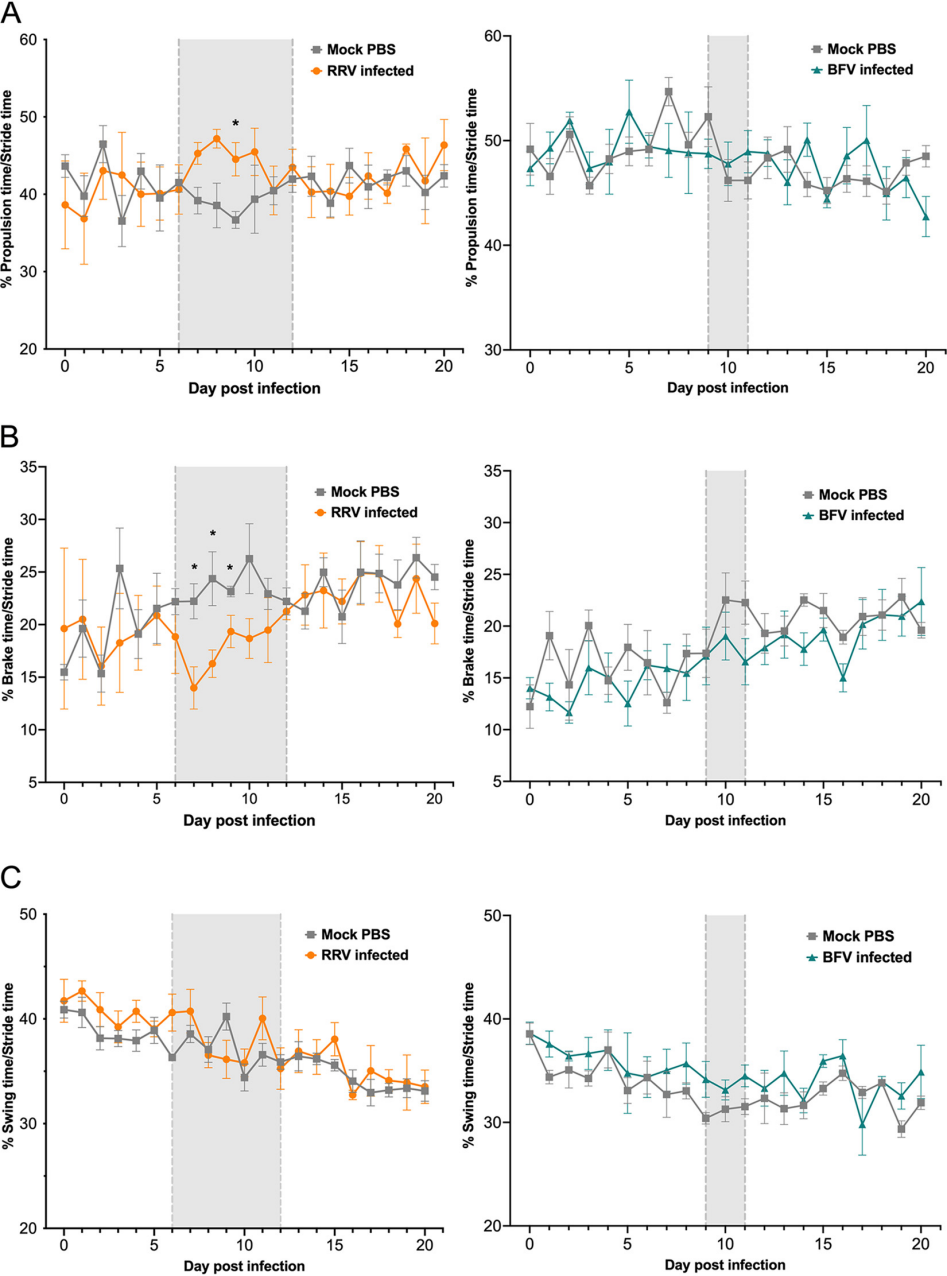
RRV-infected mice show significant changes in temporal gait parameters at peak acute disease compared to mock-infected mice. Percent propulsion/stride (percentage of time that the propulsion time contributes to one complete stride cycle), percent brake/stride (percentage of time that the brake time contributes to one complete stride cycle), and percent swing/stride (percentage of time that the swing time contributes to one complete stride cycle) were analyzed in the right hindlimbs of RRV-, BFV-, and mock-infected mice (*n *= 5) using the DigiGait apparatus. The values are the means ± standard errors of the means. Percent propulsion/stride, percent brake/stride, and percent swing/stride were analyzed using the nonparametric Mann-Whitney test (*, *P < *0.05). The gray background bound by gray dashed lines indicates the period during which RRV-infected mice received an average clinical disease score of ≥2 and BFV-infected mice received an average clinical disease score of ≥1.

**FIG 6:**
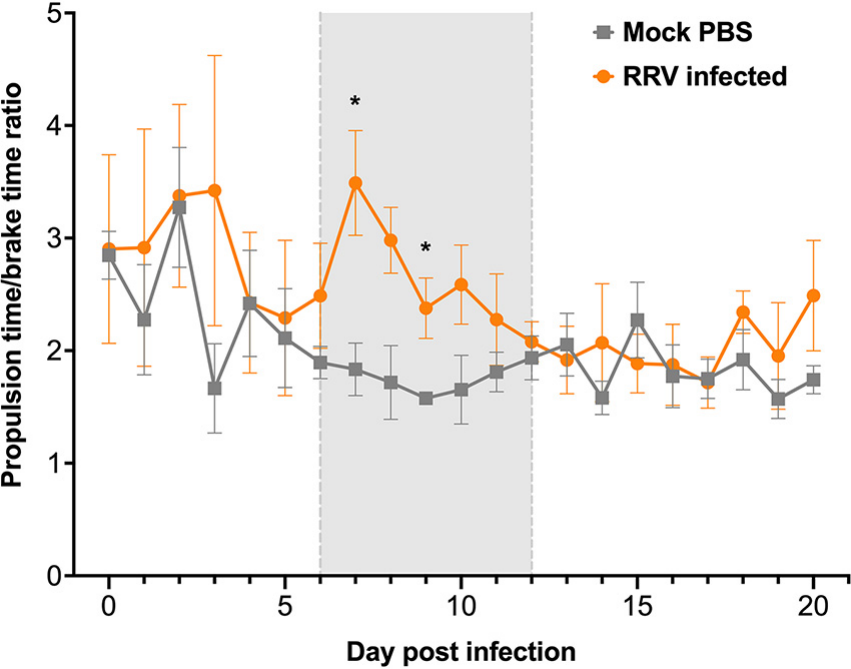
Increased propulsion time/brake time ratio in RRV-infected mice. Propulsion time and brake time were analyzed in the right hindlimbs of RRV- and mock-infected mice (*n *= 5) using the DigiGait apparatus. Values are the means ± standard errors of the means. The propulsion time/brake time ratio was analyzed using the nonparametric Mann-Whitney test (*, *P < *0.05). The gray background bound by gray dashed lines indicates the period during which RRV-infected mice received an average clinical disease score of ≥2.

Stride, stance, swing, brake, and propel times were measured in the right hindlimb to detect temporal changes in the gait of arthritogenic alphavirus-infected mice. There was no significant difference in temporal gait parameters between mock- and BFV-infected mice (Fig. 5). However, RRV-infected mice showed higher values for percent propulsion/stride than mock-infected mice during peak disease, with the increase reaching statistical significance at 9 dpi (Fig. 5A). Concurrently, the percent brake/stride decreased significantly in RRV-infected mice early during peak disease (7, 8, and 9 dpi) (*P < *0.05) (Fig. 5B). No difference in percent swing/stride was detected between mock- and RRV-infected mice over the course of infection (Fig. 5C). Thus, temporal changes in gait were detected only during the stance time (the part of the stride in which the paw remains in contact with the belt). Brake and propulsion times were analyzed to confirm their contribution to stance time. RRV-infected mice displayed a decrease in brake time (time between initial paw contact and maximum paw contact with the belt) and an increase in propulsion time (time between maximum paw contact and the start of the swing phase) during peak disease compared to mock-infected mice. Accordingly, RRV-infected mice had a significantly higher propulsion time/brake time ratio than mock controls early during peak disease (7 and 9 dpi) (*P < *0.05) (Fig. 6). These results demonstrate that both spatial and temporal gait parameters are significantly altered in RRV-infected mice during peak acute disease.

## DISCUSSION

The established RRV and BFV murine models of acute arthritogenic alphavirus disease accurately recapitulate human clinical disease. Infected mice develop detectable viremia, and infectious virus disseminates to the skeletal muscles and joints, causing an inflammatory response that results in transient myositis and arthralgia. Both models are routinely used to elucidate the immunopathology of alphavirus infection and trial novel therapeutics (8, 21, 22). Using the DigiGait system, we sought to objectively identify changes in gait parameters that could serve as reliable markers of acute alphavirus disease. To our knowledge, this is the first time that DigiGait has been used to characterize gait in mice with infectious arthritis, particularly virus-induced arthritis.

Both RRV- and BFV-infected mice required intervention, to various degrees, to reach a running speed of 25 cm/s on the DigiGait apparatus. A constant speed of 25 cm/s was used throughout the experiments to analyze gait over the course of infection. Interventions to stimulate running in infected mice, including turning the DigiGait treadmill on and off, were required in the lead-up to, during, and immediately following peak disease. Typically, RRV-infected mice with a clinical disease score of >1 required some level of intervention to reach 25 cm/s. The need for intervention also aligned with the significantly reduced weight gain in RRV-infected mice compared to mock controls. In contrast to RRV-infected mice, BFV-infected mice required minimal intervention to reach 25 cm/s over the course of infection, reflecting the milder disease profile of the BFV model of infection.

DigiGait analysis of RRV-infected mice detected significant changes in both temporal and spatial gait parameters compared to mock-infected mice. The overall hindlimb paw angle increased significantly in RRV-infected mice at peak disease compared to mock-infected mice. An increase in the overall hindlimb paw angle at peak disease aligns with the observable clinical signs of RRV disease in mice at peak disease, which include splayed hindlimbs. Increased paw angle is a feature of other models of arthritic disease, including antigen-induced arthritis, where outward rotation of the paws correlated with joint destruction (23). Toe-out angles also typically increase during bilateral injury in rodent models, and larger toe-out angles can indicate a compensatory gait pattern used to overcome instability (24, 25).

The overall hindlimb paw area tended to decrease during acute RRV disease in mice, with a significant decrease compared to mock controls at peak disease and during the resolution of disease. Reduced print area has been consistently observed in various rodent models of arthritis. In models of lipopolysaccharide (LPS)- and pristane-induced arthritis, the paw area was reduced in diseased mice compared to mock controls, with correlations between reduced paw area, clinical disease signs, and histopathological measures of disease (26, 27). Studies using the DigiGait system to examine rodent models of osteoarthritis and carrageenan-induced arthritis have also detected decreased paw area coinciding with disease onset (19, 28). Decreased paw area implies greater weight on a smaller foot area, which is suggested to protect specific joints from bearing weight (28).

The percent propulsion/stride and percent brake/stride were significantly altered in RRV-infected mice early during peak acute disease. The results suggest that the right hindlimb of RRV-infected mice rapidly comes into maximum contact with the belt during peak disease. These mice also required an extended period of time to fully lift the hindlimb from the belt at peak disease. These changes may indicate a weakened hindlimb or compensatory gait. That these temporal gait parameters were modified early during the period of peak disease suggests that acute RRV disease most significantly impacts gait during the onset of disease. Accordingly, the onset period of the disease (6 to 9 dpi) in RRV-infected mice is associated with peak expression of proinflammatory mediators, a sharp influx of inflammatory monocytes, and myositis in skeletal muscle (14). Known changes in the immunopathology of the joints and muscles are likely to contribute to gait modifications in RRV-infected mice. A significant enhancement of the propulsion times of affected legs was detected in a murine model of transforming growth factor β1 (TGFβ1)-induced osteoarthritis (29). Carrageenan-injected rats also showed significantly higher values for percent propulsion/stride than the vehicle controls in diseased limbs (19). Gait has been suggested to constitute an alternative and potentially clinically relevant measure of pain due to inflammation (18). Thus, the changes in gait arising during acute infection in mice may indicate compensatory mechanisms used to alleviate pain arising due to RRV-induced inflammation.

In contrast to RRV infection, no significant difference in gait parameters was detected in BFV-infected mice compared to mock controls. These results suggest that changes in gait are not a feature of acute BFV infection in mice, which exhibit mild musculoskeletal pathology. It is possible that the DigiGait system lacked sensitivity to detect significantly altered gait in BFV-infected mice, potentially due to the mild disease presentation in this model. Previous studies attest to the limitations of DigiGait and similar systems to accurately define complex changes that occur in the arthritic joints of small animals (19). Thus, it is unclear whether overt signs of disease are required for the DigiGait system to detect significant changes in gait or whether more severe disease results in changes in gait that are detected by DigiGait. It is important, then, to note that changes in gait in RRV-infected mice were detected only at peak disease, when clinical signs of disease are most obvious and levels of markers of immunopathogenesis are at their highest (14).

The DigiGait system therefore offers an objective measure of alterations in gait in RRV-infected mice. The correlation between significant gait changes, clinical signs of disease, and the degree of intervention required for mice to run on the DigiGait apparatus provides evidence for the potential animal welfare benefits of DigiGait, not requiring increased animal numbers to obtain significant results (19). Additional benefits of DigiGait include the removal of labor-intensive blind scoring and subjective interpretation of clinical signs. DigiGait will assist in determining the therapeutic value of novel drugs to treat arthritogenic alphavirus disease and reduce reliance on experienced users to score disease by observing mouse behavior. We have used DigiGait to confirm the debilitating effect of alphavirus infection on mice and provide a new model of RRV infection that measures gait parameters as objective manifestations of the progression of acute disease. DigiGait will likely have utility in measuring gait in other small-animal models of infectious arthritis, particularly those that develop severe hindlimb weakness such as that observed in RRV-infected mice.

## MATERIALS AND METHODS

### Viruses.

The T48 prototype strain of Ross River virus (RRV) (isolated in Townsville, Queensland, Australia, in 1959 [30]) was kindly provided by Richard Kuhn, Purdue University, and generated via *in vitro* transcription of a SacI-linearized pRR64 plasmid encoding the full-length T48 clone. The BFV2193 prototype Barmah Forest virus (BFV) strain was used. All viruses had similar passage histories (31, 32). Briefly, following initial propagation in suckling mouse brain, virus was expanded *in vitro* in Vero cells (not exceeding 3 passages). Viruses were titrated by a plaque assay on Vero cells, as described previously (21), and diluted to the required concentration (10^4^ PFU) in sterile phosphate-buffered saline (PBS) for animal experiments.

### Mice and infections.

Animal experiments were approved by the Animal Ethics Committee of Griffith University (GLY12/18 and GLY02/18). All procedures involving animals conformed to the *Australian Code of Practice for the Care and Use of Animals for Scientific Purposes* (33). Wild-type C57BL/6 mice were obtained from the Animal Resource Centre (Perth, Australia). Mice were housed in the Bioscience Resource Facilities of Griffith University (Gold Coast, Australia). For the RRV and BFV mouse models, 21-day-old C57BL/6 female mice (*n *= 5) were inoculated subcutaneously below the right forelimb with 10^4^ PFU of RRV or BFV in 50 μl, and mock-infected mice were inoculated with 50 μl of PBS diluent alone. Mice were weighed and scored for disease daily. RRV and BFV disease scores were assessed visually based on strength and hindlimb dysfunction using the following scale, as described previously (21): 0 for no disease signs; 1 for ruffled fur, 2 for very mild hindlimb weakness, ruffled fur, and some lethargy; 3 for mild hindlimb weakness, starting to show signs of delicate walking, and an increase in lethargy; 4 for moderate hindlimb weakness, mild dragging of hindlimbs, and limited gripping ability (labored walking pattern with splayed hind legs); and 5 for severe hindlimb weakness/dragging, limited movement and stride of hind legs when walking, and moderate lethargy.

### Histological analysis.

At 10 dpi, mouse quadriceps were dissected and fixed in 4% paraformaldehyde, followed by paraffin embedding. Samples were cut into 5-μm-thick sections and stained with hematoxylin and eosin. Images were taken using an Aperio AT2 digital whole-slide scanner (Leica).

### Gait analysis.

Gait analysis was performed using a DigiGait imaging system (Mouse Specifics Inc., Boston, MA). This system allows mice, and other small animals, to walk or run on a motor-driven transparent treadmill belt. Below the belt, a mounted video camera records ventral images of animals during locomotion (Fig. 3A). Images are automatically analyzed to create a digital paw print and dynamic gait signals (Fig. 3B). The generated signals describe the posture and kinematics of the animals and reflect strength, balance, and coordination. Spatial (Fig. 3C) and temporal (Fig. 3D) gait parameters can be measured from the generated signals. Mice were trained on the DigiGait treadmill at speeds of 10 to 25 cm/s for a maximum of 1 min per mouse for 5 days prior to infection to become acclimatized to the apparatus. All mice were compliant with the DigiGait apparatus prior to infection. Mice were recorded in the same field of view for at least 10 consecutive gait cycles at 25 cm/s. Recordings were performed at the same time (10:00 a.m.) daily, with background noise and lighting standardized across all time points. A speed of 25 cm/s was chosen after trialing lower running speeds in pilot experiments. At the point of infection, mice were 21 days old and by the conclusion of the experimental procedure had gained >100% body weight. A speed of 25 cm/s ensured that all mice, mock and infected, were able to consistently ambulate throughout the experiment.

Both RRV- and BFV-infected mice demonstrated difficulty in reaching a running speed of 25 cm/s around the period of peak acute disease. Mice were scored on the degree of intervention required to stimulate running at 25 cm/s using the scale described in Table 1.

**TABLE 1:** DigiGait intervention score scale

Intervention score	Description of intervention
0	Reaches 25 cm/s without any intervention
1	Reaches 25 cm/s after <5 on/off cycles of the DigiGait treadmill
2	Reaches 25 cm/s after <2 min of habituation to the apparatus followed by >5 on/off cycles of the DigiGait treadmill
3	Reaches 25 cm/s after 2–5 min of habituation to the apparatus followed by >5 on/off cycles of the DigiGait treadmill
4	Reaches 25 cm/s after >5 min of habituation to the apparatus followed by >5 on/off cycles of the DigiGait treadmill
5	Difficulty reaching 25 cm/s after >5 min of habituation to the apparatus followed by >5 on/off cycles of the DigiGait treadmill and had to stimulate running at 25 cm/s by starting at a lower speed (15–20 cm/s)
6	Unable to reach 25 cm/s

### Statistics.

All results are presented as the means ± standard errors of the means. Mouse weight was analyzed using two-way analysis of variance (ANOVA) with a Bonferroni *post hoc* test. Spatial and temporal gait parameters were analyzed using the nonparametric Mann-Whitney test. All statistical analyses were performed with GraphPad Prism 9.1.2 software.
